# New Bur and Drill Gauge

**Published:** 1883-10

**Authors:** 


NEW BUR AND DRILL GAUGE.

Mr. Geo. E. Hodge is manufacturing a new gauge for determining the size of dental burs and drills. It is of steel, and made upon the same principles as the wire gauges in common use, being pierced with holes of a standard size, which are regularly marked from 000 to 14. It is a great convenience in ordering, as it accurately measures any kind of bur or drill.

